# Intrapleural infusion of tumor cell-derived microparticles packaging methotrexate or saline combined with pemetrexed-cisplatin chemotherapy for the treatment of malignant pleural effusion in advanced non-squamous non-small cell lung cancer: A double-blind, randomized, placebo-controlled study

**DOI:** 10.3389/fimmu.2022.1002938

**Published:** 2022-10-05

**Authors:** Xiaorong Dong, Yu Huang, Tienan Yi, Chunhong Hu, Quanli Gao, Yuan Chen, Jing Zhang, Jianhua Chen, Li Liu, Rui Meng, Sheng Zhang, Xiaofang Dai, Shihong Fei, Yang Jin, Ping Yin, Yanping Hu, Gang Wu

**Affiliations:** ^1^ Cancer Center, Union Hospital, Tongji Medical College, Huazhong University of Science and Technology, Wuhan, China; ^2^ Department of Oncology, Xiangyang Central Hospital, Affiliated Hospital of Hubei University of Arts and Science, Xiangyang, China; ^3^ Department of Oncology, The Second Xiangya Hospital of Central South University, Changsha, China; ^4^ Department of Immunotherapy, Affiliated Cancer Hospital of Zhengzhou University & Henan Cancer Hospital, Zhengzhou, China; ^5^ Department of Oncology, Tongji Hospital, Tongji Medical College, Huazhong University of Science and Technology, Wuhan, China; ^6^ Department of Thoracic Oncology, Hubei Cancer Hospital, Tongji Medical College, Huazhong University of Science and Technology, Wuhan, China; ^7^ Thoracic Medicine Department, Hunan Cancer Hospital, Changsha, China; ^8^ Department of Respiratory and Critical Care Medicine, Union Hospital, Tongji Medical College, Huazhong University of Science and Technology, Wuhan, China; ^9^ Department of Epidemiology and Biostatistics, School of Public Health, Tongji Medical College, Huazhong University of Science and Technology, Wuhan, China

**Keywords:** intrapleural infusion, methotrexate, microparticles, malignant pleural effusion, non-squamous non-small cell lung cancer

## Abstract

**Background:**

Preclincal studies showed the promising efficacy of tumor cell-derived microparticles packaging methotrexate (TMPs-MTX) to treat advanced non-squamous non-small cell lung cancer (NSCLC) with malignant pleural effusion (MPE).

**Methods:**

This randomized, double-blind, placebo-controlled study was conducted at six hospitals in China from 20 July 2015 to 25 April 2019. Patients newly diagnosed with non-squamous NSCLC with MPE were randomly assigned to receive TMPs-MTX (group A) or saline (group B). Patients in both groups received pemetrexed (500 mg/m^2^ d1) and cisplatin (75 mg/m^2^ in total for d1-d2). Intrapleural infusion (50 mL saline containing 5 units of TMPs-MTX per perfusion, once every 48 hours, six total perfusions) was initiated on day 5 after pemetrexed-cisplatin chemotherapy. The primary outcome was the objective response rate (ORR) of MPE. Secondary outcomes included the ORR of target lesions, progression-free survival (PFS), overall survival (OS), toxicity, and pleural fluid properties.

**Results:**

A total of 86 patients were enrolled in this study and randomly assigned to either group A or group B. Of these, 79 patients were evaluable for response. The ORR of MPE in group A was significantly higher than that in group B (82.50% vs. 58.97%, *P* = 0.0237). The ORR of target lesions was 25.64% in group A and 20.51% in group B (*P* = 0.5909), respectively. With a median follow-up time of 18.8 months, median PFS were 6.4 (95% CI, 4.5-12.3) months in group A and 7.3 (95% CI, 6.1-10.4) months in group B (*P* = 0.6893), and median OS were 19.9 (95% CI, 17.1-28.5) months and 17.5 (95% CI, 11.6-25.0) months (*P* = 0.4500), respectively. The incidence rates of adverse events were similar in the two groups. The most common treatment-related adverse events were chemotherapy-induced toxicities, including fever, gastrointestinal reactions, hepatic dysfunction, and leukopenia.

**Conclusion:**

Intrapleural infusion of TMPs-MTX combined with pemetrexed-cisplatin chemotherapy is safe and effective against MPE in patients with advanced non-squamous NSCLC.

**Clinical trial registration:**

http://www.chictr.org.cn (ChiCTR-ICR-15006304).

## Introduction

Malignant pleural effusion (MPE) is common among patients with lung cancer, who account for approximately 30%–40% of MPE cases (1). MPE symptoms include chest distress, shortness of breath, palpitation, pain, and inability to lie prostrate. These symptoms further impact the quality of life of patients with lung cancer (2–4). The prognosis of MPE is poor, with a median survival time of 3 to 12 months (5). MPE prognosis is even worse in patients with lung cancer (5). MPE treatment methods include systemic chemotherapy, molecular targeted therapy such as tyrosine kinase inhibitors, immunotherapy and locoregional therapies (6). Locoregional MPE treatment involves the local perfusion of talc, chemotherapeutic agents, biological agents, and anti-angiogenic drugs into the pleural cavity (7–10). As there are no standardized locoregional therapies for MPE, systemic therapies and drainage through the indwelling pleural catheter are the main treatment methods. New therapeutic approaches are urgently needed for the treatment of MPE.

Microparticles (MPs) are extracellular vesicles with a size ranging between 100 and 1,000 nm. These vesicles are shed by direct budding of the cell membrane under physiological or pathological conditions. MPs regulate the communication between cells by transferring signaling molecules (proteins, lipids, nucleic acids) from donor cells to recipient cells (11). Tumor cells are able to release extracellular vesicles labeled as tumor MPs. These MPs are promising natural carriers to deliver chemotherapeutic drugs or oncolytic viruses to tumor cells (12, 13). Tumor cell-derived MPs (TMPs) can act as a cell-free tumor vaccine and stimulate dendritic cells *via* cGAS/STING signaling (14, 15). Incorporating drugs such as methotrexate (MTX) into TMPs may yield chemo-immunotherapeutic, dual-functional MPs.

TMPs packaging methotrexate (TMPs-MTX) have been proved to be safe and effective in killing tumor cells and reversing drug resistance (12, 16, 17). The exploratory clinical study showed that TMPs-MTX alleviated MPE in patients with lung cancer by modulating the pleural immune microenvironment (17). Based on the potential benefit of TMPs-MTX, we conducted a multicenter, randomized clinical trial to investigate the efficacy and safety of TMPs-MTX combined with pemetrexed-cisplatin in patients with MPE and advanced non-squamous non-small cell lung cancer (NSCLC).

## Patients and methods

### Study design and participants

This multicenter, randomized, double-blind, placebo-controlled study was carried out in six hospitals from 20 July 2015 to 25 April 2019. A total of 86 patients with MPE were enrolled. Eligible patients were 18–70 years old and were newly diagnosed with advanced non-squamous NSCLC and MPE. All patients had malignant cells in the pleural fluid. All study subjects had Karnofsky performance status (KPS) scores ≥ 70. Exclusion criteria were as follows: prior treatment with chemotherapy or intrapleural infusion, pregnancy, lactation, history of drug allergies or allergic constitution, and severe underlying diseases (e.g., cardiac and pulmonary failure, hepatic dysfunction, and renal dysfunction). The study was conducted in compliance with the Good Clinical Practice principles and the Declaration of Helsinki. All patients provided informed consent. The study was approved by the Ethics Committee of Tongji Medical College of Huazhong University of Science and Technology and was registered on the Chinese Clinical Trial Registry (ChiCTR-ICR-15006304) on April 20, 2015.

### Randomization and treatment

This was a randomized block study, with six patients per block and an allocation ratio of 1:1 in the microparticles group and the placebo group. The statisticians used SAS software to generate random number tables and the investigators were blinded to treatment allocation. Patients were randomly assigned (1:1) to receive TMPs-MTX or 0.9% saline. Patients in both groups received the same systemic chemotherapy regimen (pemetrexed 500 mg/m^2^ d1, cisplatin 75 mg/m^2^ in total for d1–d2). The indwelling pleural catheter for intrapleural infusion was inserted as per standard clinical practice. The first intrapleural infusion (50 mL per dose) was conducted at day 5 post-chemotherapy, once every other day, six times consecutively. Patients in group A were treated with 50 mL saline containing 5 units of TMPs-MTX (5 μg methotrexate/1 × 10^7^ vesicles/unit). Patients in Group B were treated with 50 mL saline as a control.

TMPs-MTX (manufacturers specifications: 50 mL/bag) were dissolved in 0.9% sodium chloride injection solution (50 mL/bag; Baite company, Shanghai, China; approval number: GuoYaoZhunZi-H19994067) so that each dose contained 5 units of methotrexate vesicles (5 μg methotrexate/1 × 10^7^ vesicles/unit). The solution was kept at 2–8°C and was returned to room temperature before use.

### Outcomes

Objective response rate (ORR) for MPE was the primary study outcome. Treatment efficacy was evaluated using the World Health Organization (WHO) evaluation criteria (18). The volume of pleural effusion was measured on computed tomography (CT) layer by layer using volume rendering in the American general post-processing workstation (GE Advance Workstation 4.5), and was visualized using post-processing software. Changes in the pleural effusion before and after treatment were determined. Complete remission (CR) was defined as complete resolution of pleural effusion for at least four weeks. Partial remission (PR) was defined as > 50% reduction in pleural effusion for at least four weeks, and stable disease (SD) was defined as < 50% reduction in pleural effusion with partial remission of clinical symptoms. No change (NC) was defined as no significant decrease or increase in the volume of MPE. The ORR of pleural effusion was calculated as CR + PR.

Secondary outcomes included the ORR of target lesions, progression-free survival (PFS), overall survival (OS), KPS score, the variation in pleural fluid properties, and treatment toxicity. Tumor markers and other biomarkers in the blood and pleural effusions were assessed before and after treatment. Tumor response was evaluated by the response evaluation criteria in solid tumors (RECIST 1.1.) (19). The target lesions met the definition of measurability as described in RECIST 1.1, including primary lung lesions, lymph nodes and metastases. The ORR of target lesions was defined as the combined proportion of patients with CR or PR. PFS was calculated from the first day of chemotherapy to the date of disease progression. OS was defined from the first day of chemotherapy to death due to any cause. The severity of adverse events was scored using the Common Terminology Criteria for Adverse Events Version 5.0 (CTCAE v5.0).

### Statistical analysis

Categorical variables were compared using either the chi-square test or the Wilcoxon rank-sum test. Numerical variables were compared using the independent sample *t*-test. A superiority trial was conducted to compare the efficacy of the treatment over the placebo. Treatment efficacy was compared using the Cochran-Mantel-Haensel method to adjust for the central effect. The chi-square test or the Fisher exact test was used to compare adverse events between the two groups and describe the changes in laboratory markers before and after treatment. Two-sided *P*-values < 0.05 were considered statistically significant. Statistical analyses were conducted using SAS version 9.4 (SAS Institute).

## Results

### Patient characteristics

**Figure 1:**
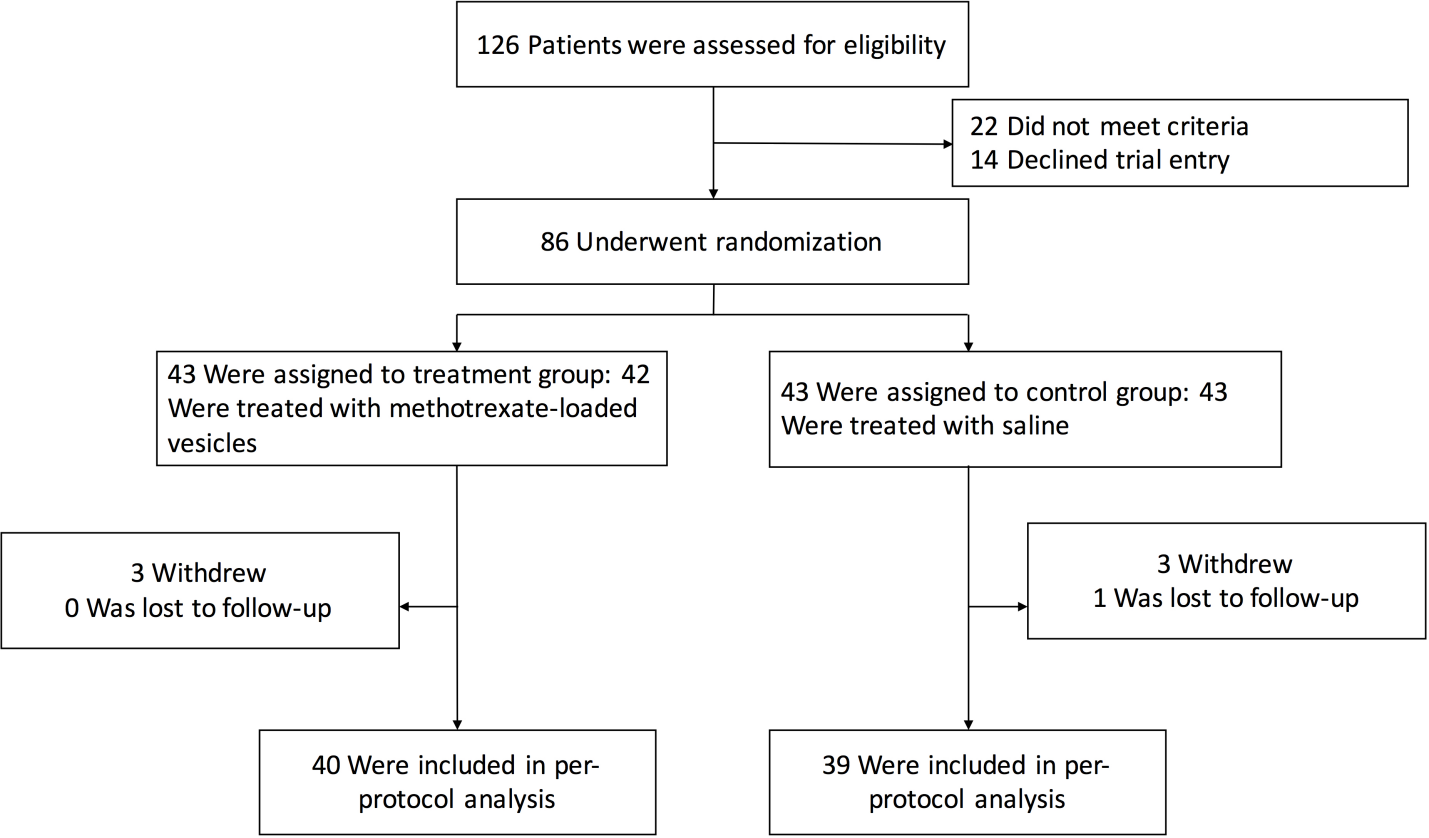
Trial profile.

This study included 86 patients with advanced non-squamous NSCLC and MPE. Patients in group A (n = 43) were treated with TMPs-MTX, and patients in group B (n = 43) received saline (**Figure 1**). The full analysis set and per-protocol set consisted of 79 (91.86%) patients who completed their treatment and attended all follow-up visits. Six patients withdrew and one patient was lost to follow-up. Therefore, seven patients (three from group A and four from group B) were excluded from our analyses (**Figure 1**). No significant differences in the baseline characteristics (age, gender, smoking history, Eastern Cooperative Oncology Group (ECOG) Performance Status, histological type, baseline volume of pleural effusion, metastatic sites and gene mutations) were observed between the two groups (*P* > 0.05; **Table 1**).

**Table 1:** Comparison of clinical characteristics between groups.

**Clinical Characteristic**	**Microparticles group** **n = 40 (%)**	**Placebo group** **n = 39 (%)**	***p***
**Sex**			
Male	16 (40.00)	23 (58.97)	0.0917
Female	24 (60.00)	16 (41.03)	
**Age (years)**			
Median	53.50	59.00	
<65	33 (82.50)	31 (79.49)	0.7328
≥65	7 (17.50)	8 (20.51)	
**Smoking status**			
Never smoker	13 (32.50)	18 (46.15)	0.2093
Ever smoker	26 (65.00)	20 (51.28)	
NA	1 (2.50)	1 (2.57)	
**ECOG performance status**			
1	34 (85.00)	34 (87.18)	0.7797
2	6 (15.00)	5 (12.82)	
**Histological type**			
Adenocarcinoma	40 (100.00)	39 (100.00)	–
Others	0 (0.00)	0 (0.00)	
**Baseline volume of pleural effusion**			
<1000ml	29 (72.50)	22 (56.41)	0.1350
≥1000ml	11 (27.50)	17 (43.59)	
**Metastatic sites**			
Brain	8 (20.00)	8 (20.51)	
Bone	18 (45.00)	12 (30.77)	0.5421
Others	6 (15.00)	8 (20.51)	
**T stage**			
T1	4 (10.00)	4 (10.26)	0.9532*
T2	9 (22.50)	8 (20.51)	
T3	5 (12.50)	3 (7.69)	
T4	17 (42.50)	17 (43.59)	
Tx	5 (12.50)	7 (17.95)	
**N stage**			
N0	0 (0.00)	2 (5.13)	0.0951*
N1	5 (12.50)	1 (2.56)	
N2	18 (45.00)	18 (46.15)	
N3	15 (37.50)	11 (28.21)	
Nx	2 (5.00)	7 (17.95)	
***EGFR* mutation**			
Yes	15 (37.50)	15 (38.46)	0.5275
No	18 (45.00)	13 (33.33)	
Not-test	7 (17.50)	11 (28.21)	
**ALK status**			
Positive	1 (2.50)	2 (5.13)	0.7937
Negative	19 (47.50)	13 (33.33)	
Not-test	20 (50.00)	24 (61.54)	

*Fisher’s exact test.

ECOG, Eastern Cooperative Oncology Group; EGFR, epidermal growth factor receptor; ALK, anaplastic lymphoma kinase; NA, not available.

### Primary outcome

Among the 40 patients in group A, there were 10 CR cases, 23 PR cases, 1 SD case, and 6 NC cases. Among the 39 patients in group B, there were 6 CR cases, 17 PR cases, 8 SD cases, and 8 NC cases. The ORR for MPE in group A was significantly higher than that in group B (82.50% vs. 58.97%; *P* = 0.0237; **Table 2**). These results suggest that TMPs-MTX alleviate MPE in patients with advanced non-squamous NSCLC.

**Table 2:** Efficacy of malignant pleural effusion and target lesions.

	Microparticles group (n = 40)	Placebo group(n = 39)
Malignant pleural effusion		
ORR	82.50%	58.97%
Target lesions		
ORR	25.64%	20.51%
DCR	97.44%	92.31%

ORR, objective response rate; DCR, disease control rate.

### Secondary outcomes

Among the 39 evaluable patients of target lesions in group A, there were 0 CR cases, 10 PR cases, 28 SD cases, and one PD case. The ORR was 25.64%, and the disease control rate (DCR; CR+PR+SD) was 97.44%. Among the 39 patients in group B, the numbers of patients with CR, PR, SD, and PD were 0, 8, 28, and 3, respectively. The ORR and DCR were 20.51% and 92.31%, respectively (**Table 2**). Both ORR and DCR in group A were higher than those in group B, although their differences were not statistically significant (*P* = 0.5909 and *P* = 0.6077, respectively).

**Figure 2:**
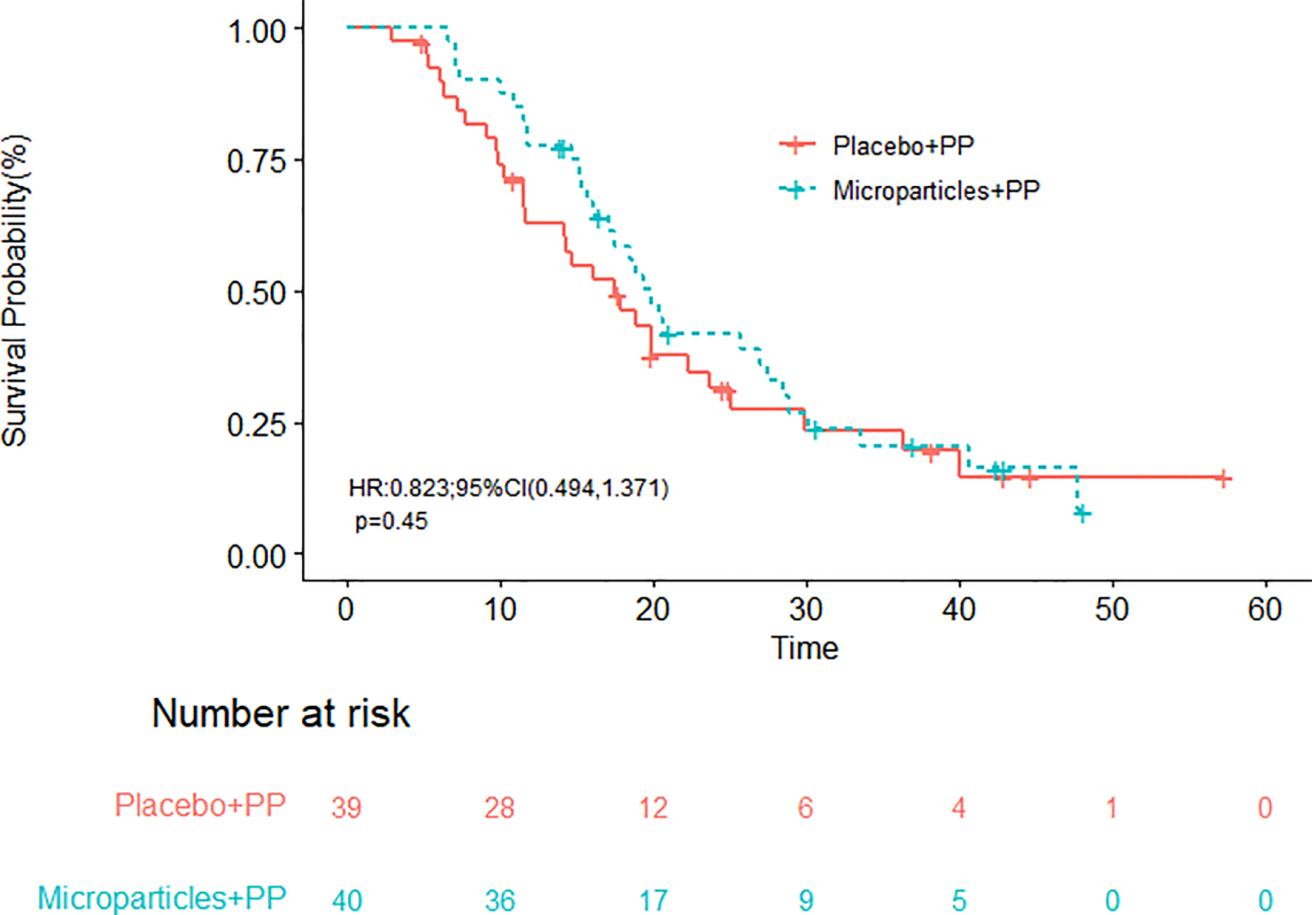
Overall survival of patients receiving the tumor cell-derived microparticles packaging methotrexate (TMPs-MTX) or saline combined with pemetrexed-cisplatin (PP) chemotherapy.

**Figure 3:**
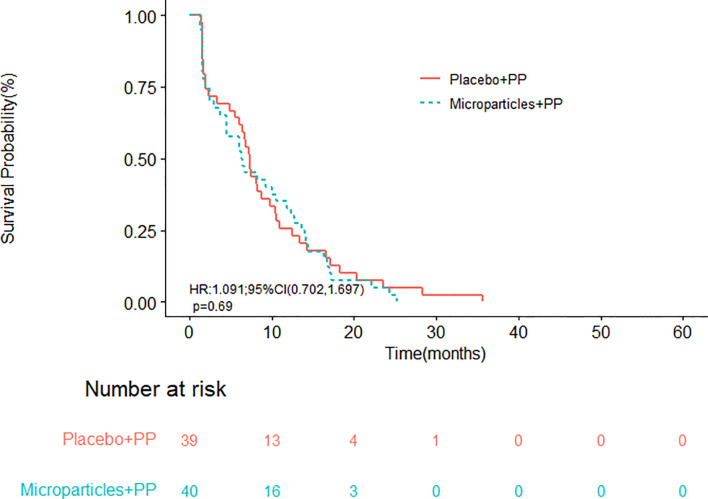
Progression-free survival of patients receiving the tumor cell-derived microparticles packaging methotrexate (TMPs-MTX) or saline combined with pemetrexed-cisplatin (PP) chemotherapy.

With a median follow-up time of 18.8 months, the median OS in group A and group B were 19.9 (95% CI, 17.1-28.5) and 17.5 (95% CI, 11.6-25.0) months, respectively (**Figure 2**); the difference in OS was not statistically significant (*P* = 0.4500). The half-year OS (100.00% vs. 89.74%) and one-year OS (77.50% vs. 58.97%) rates in group A were higher than those in group B, although their differences were not statistically significant. The median PFS were 6.4 (95% CI, 4.5-12.3) months in group A and 7.3 (95% CI, 6.1-10.4) months in group B (*P* = 0.6893; **Figure 3**).

There was no significant difference in the KPS scores before and after treatment between the two groups (*P* >0.05). Moreover, we found no significant differences in the blood levels of the tumor markers CEA, CYFRA21-1, CA125, and CA19-9 between the two groups (*P* >0.05, **Supplementary Table 1**). Furthermore, there were no statistically significant differences in Rivalta test parameters (pleural fluid routine) between the two groups (**Supplementary Table 2**). Similarly, no significant differences in the levels of total protein, glucose, lactate dehydrogenase, and CEA in the pleural fluid were observed between the two groups (**Supplementary Table 3**).

### Adverse effects

A total of 56 adverse events (cumulative number: 194 cases) was reported, with 38 drug-related adverse events (cumulative number: 124 cases). 30 adverse events were reported in group A, and 26 adverse events were reported in group B. No statistically significant differences were observed in the incidence of adverse events between the two groups (*P *= 0.4647). There were 21 (50%) drug-related adverse events in group A and 17 (39.53%) drug-related adverse events in group B. The differences in the rates of drug-related adverse events between the two groups were also not statistically significant (*P* = 0.4891). 7 serious adverse events were reported: 4 in group A and 3 in group B. The incidence of serious adverse events did not differ significantly between the two groups. One drug-related serious adverse event was reported in group A; this adverse event was hepatic dysfunction (grade 3). The most common treatment-related adverse events were chemotherapy-induced toxicity, including fever, gastrointestinal reactions, hepatic dysfunction, leukopenia, asthenia, anemia, and hypoalbuminemia. The incidence rates of fever and hepatic dysfunction were slightly higher in group A than in group B (**Table 3**).

**Table 3:** Adverse events.

Adverse events	Microparticles group (n = 40)				Placebo group (n = 39)			
	Grade 1	Grade 2	Grade 3	Grade 4	Grade 1	Grade 2	Grade 3	Grade 4
Pyrexia	7 (18%)	2 (5%)	1 (3%)	0	5 (13%)	2(5%)	0	0
Vomiting	4 (10%)	0	1 (3%)	0	4 (10%)	1 (3%)	0	0
Fatigue	4 (10%)	0	0	0	3 (8%)	1(3%)	1 (3%)	0
Nausea	3 (8%)	1 (3%)	0	0	5 (13%)	3 (8%)	1 (3%)	0
Thoracalgia	2 (5%)	1 (3%)	0	0	0	0	0	0
Chest stuffiness	0	0	0	0	4 (10%)	1 (3%)	0	0
Leukopenia	3 (8%)	1 (3%)	0	0	5 (13%)	3 (8%)	1 (3%)	0
Infection	0	1 (3%)	0	0	0	1 (3%)	2 (5%)	0
Thrombus	0	0	0	0	0	1 (3%)	0	0
Atrial Fibrillation	0	0	0	0	0	1 (3%)	0	0
Pain	0	2 (5%)	0	0	0	2 (5%)	0	0
Hypertension	0	1(3%)	0	0	0	1 (3%)	1 (3%)	0
Anemia	3 (8%)	2 (5%)	0	0	2 (5%)	1 (3%)	0	0
Abnormal liver function	1 (3%)	4 (10%)	1 (3%)	0	4 (10%)	0	0	0
Constipation	1 (3%)	1 (3%)	0	0	2 (5%)	0	0	0
Hypoproteinemia	3 (8%)	1 (3%)	0	0	2 (5%)	1 (3%)	0	0
Cough	1 (3%)	0	0	0	0	1 (3%)	1 (3%)	0
Dyspnoea	0	0	0	0	0	0	1 (3%)	0
Urine leukocytosis	0	1 (3%)	0	0	0	0	0	0
Erythra	1 (3%)	1 (3%)	0	0	0	1 (3%)	0	0

## Discussion

In this study, we evaluated the efficacy of the TMPs-MTX in the treatment of MPE in patients with advanced non-squamous NSCLC. We found that, compared with the control treatment, TMPs-MTX significantly alleviated MPE in patients with NSCLC and revealed good safety. Most adverse events were manageable. Although no significant improvements were observed in the secondary outcomes, including the ORR of target lesions, OS and PFS. Intrapleural infusion of TMPs-MTX still provides a new locoregional treatment option for patients with MPE.

Indwelling pleural catheters are often used to control MPE symptoms in patients with solid tumors (20–23). However, this method increases the risk of certain infections blockage, pneumothorax and catheter track metastasis (24, 25). Furthermore, no significant difference in the relief of breathlessness was observed between indwelling catheter alone and talc administration through an indwelling pleural catheter (26). In the previous studies of MPE, patients with different types of cancers were recruited, including lung cancer, breast cancer, mesothelioma, ovarian cancer and so on. As the systemic treatment regimens were non-uniform among these previous studies, the therapeutic effect of systemic treatment on MPE could not be comparable, and this may lead to research bias. In the management of MPE caused by NSCLC, bevacizumab intrapleural infusion showed higher response rate than intravenous infusion (27). Cytotoxic drugs such as nedaplatin or cisplatin were also infused intrapleurally for controlling MPE, but only 50%–60% patients responded to this treatment (28). These cytotoxic drugs might cause gastrointestinal side effects or other toxicities (28). Debulking surgery and hyperthermic intrathoracic chemotherapy achieved encouraging outcome in the treatment of selected patients with NSCLC and MPE (29). But the quality of evidence is still weak to confirm the effectiveness of this treatment (29). Lung cancer is one of the most common causes of MPE. The median survival time of lung cancer patients with pleural effusion was found to be only 74 days (5). Thus, novel therapeutic approaches for the treatment of MPE in patients with NSCLC are urgently required.

A recent study by Guo et al. (17) showed that TMPs-MTX were safe and effective in 11 patients with advanced lung cancer and MPE. The ORR was 90.91%, including 4 CRs and 6 PRs. The median time of pleurodesis was seven days. Long-term follow-up revealed that 9 of 11 patients did not need further therapeutic pleural drainage until death (17). The viability of malignant cells in the MPE was also tested in previous research. CD45- cells, which were confirmed to be tumor cells by HE staining, were efficiently removed from the MPE in TMPs-MTX treated patients. However, in the saline-treated patients, the proportions of CD45- cells were not altered (30). Our study is the first randomized controlled trial evaluating the efficacy of TMPs-MTX in the treatment of MPE. We found that the combination of TMPs-MTX with pemetrexed-cisplatin significantly alleviated MPE in patients with advanced non-squamous NSCLC. However, patients in both groups received first-line pemetrexed-cisplatin chemotherapy. The same systemic therapy contributed to similar ORR of target lesions and PFS between these two groups. In addition, some patients with epidermal growth factor receptor (EGFR) and anaplastic lymphoma kinase (ALK) alterations might be treated with EGFR or ALK tyrosine kinase inhibitors (TKIs) after disease progression of chemotherapy. Therefore, the OS was also influenced by second-line treatment. Patient reported outcomes including symptoms and quality-of-life scores improvement for intrapleural infusion of TMPs-MTX might be worth exploring.

The biological mechanisms underlying the therapeutic effects of TMPs-MTX (**Supplementary Figure 1**) have been explored in recent studies (12, 16, 31). It has been reported that intrapleural infusion of TMPs-MTX significantly decreased the numbers of tumor cells and CD163^+^ macrophages in the pleural immune microenvironment. TMPs-MTX also stimulated IL-2 secretion in CD4^+^ T cells and IFN-γ secretion in CD8^+^ T cells (17). Upon methotrexate-loaded vesicle entry into the MPE, the vesicles were recognized and engulfed by tumor cells, leading to tumor cell death (17). Phagocytosis of TMPs-MTX by macrophages induced the release of CXCL1 and CXCL2, promoting neutrophil chemotaxis toward the MPE (30). These activated neutrophils enhanced the elimination of tumor cells. Moreover, activated neutrophils were reported to release web-like DNA-containing structures (i.e., neutrophil extracellular traps) to entrap pathogens and inhibit endothelial damage, thereby attenuating the inflammatory response in MPE (30).

Each unit of drug loaded microparticles contained about 5 ± 1ug of methotrexate. The earliest safe dose exploration test began with the perfusion of 3 units TMPs-MTX for each time, while 5 units were the second dose group. The previous study showed that intrapleural delivery of 5 units TMPs-MTX were safe and effective in lung cancer patients with MPE (17). The adverse reactions were only grade 1-2 (17). Therefore, the same dose of 5 units of TMPs-MTX was used in this clinical study and TMPs-MTX were well-tolerated. There were 38 drug-related adverse events in this study. Notably, we found no significant differences in the incidence of adverse events between the two groups, suggesting that TMPs-MTX were safe in patients with advanced non-squamous NSCLC.

Limitations of this study included the small sample size. And the immune related factors in pleural fluid or blood were not tested. Additionally, participant-reported health-related quality of life and symptoms in these two groups were not compared in this study. But many secondary outcomes were discussed, including KPS scores, levels of tumor markers in the blood, and pleural fluid properties. Despite these limitations, our study is one of the very few randomized controlled trials focusing on MPE in patients with advanced non-squamous NSCLC.

In conclusion, MPs might be an attractive drug delivery system and TMPs-MTX was reported to be chemo-immunotherapeutic, dual-functional in previous studies. Our findings suggest that intrapleural infusion of TMPs-MTX is an effective and safe approach to treat MPE in patients with advanced non-squamous NSCLC. MPE often require pleural intervention for symptom control. TMPs-MTX may provide a new locoregional strategy to treat malignancies with MPE.

## Data availability statement

The original contributions presented in the study are included in the article/**Supplementary Material**. Further inquiries can be directed to the corresponding author.

## Ethics statement

The studies involving human participants were reviewed and approved by the Ethics Committee of Tongji Medical College of Huazhong University of Science and Technology. The patients/participants provided their written informed consent to participate in this study.

## Author contributions

XDo (1st author) conceived and designed the study, reviewed and edited the manuscript. YuH (2nd author) interpreted the data and wrote the manuscript. TY, CH, QG, YC, JZ, LL, RM, SZ, XDa (12th author) and SF contributed to patient management. JC and YJ interpreted the data. PY conducted the statistical analysis. YaH (16th author) provided effective suggestion for treatment and modification, interpreted the data. GW was involved in the study concept and design, critical revision of the manuscript and study supervision. All authors contributed to the article and approved the final version of the manuscript.

## Funding

This study was supported by the grants from the National Key R&D Program of China (Grant No. 2019YFC1316205) and grants from Key Research and Develop Program of Hunan Province, China (Grant No. 2017WK2061) and the Key Research and Development Program of Hubei (Grant No. 2020BCA068).

## Acknowledgments

The authors thank all the patients, their families and the clinical study teams who participated in the study. We thank Yao Xiao (Department of Epidemiology and Biostatistics, School of Public Health, Tongji Medical College, Huazhong University of Science and Technology) for the assistance of statistical data analysis. The study was also sponsored by Soundny (Sheng-Qi-An) Biotech.

## Conflict of interest

The authors declare that the research was conducted in the absence of any commercial or financial relationships that could be construed as a potential conflict of interest.

The reviewer SF declared a shared parent affiliation with the author CH to the handling editor at the time of the review

## Publisher’s note

All claims expressed in this article are solely those of the authors and do not necessarily represent those of their affiliated organizations, or those of the publisher, the editors and the reviewers. Any product that may be evaluated in this article, or claim that may be made by its manufacturer, is not guaranteed or endorsed by the publisher.
