# Supplementary figures and images for: Intrapleural infusion of tumor cell-derived microparticles packaging methotrexate or saline combined with pemetrexed-cisplatin chemotherapy for the treatment of malignant pleural effusion in advanced non-squamous non-small cell lung cancer: A double-blind, randomized, placebo-controlled study
Source: Front Immunol. 2022 Oct 5;13:1002938. doi: 10.3389/fimmu.2022.1002938 (PMC9580337; doi:10.3389/fimmu.2022.1002938)

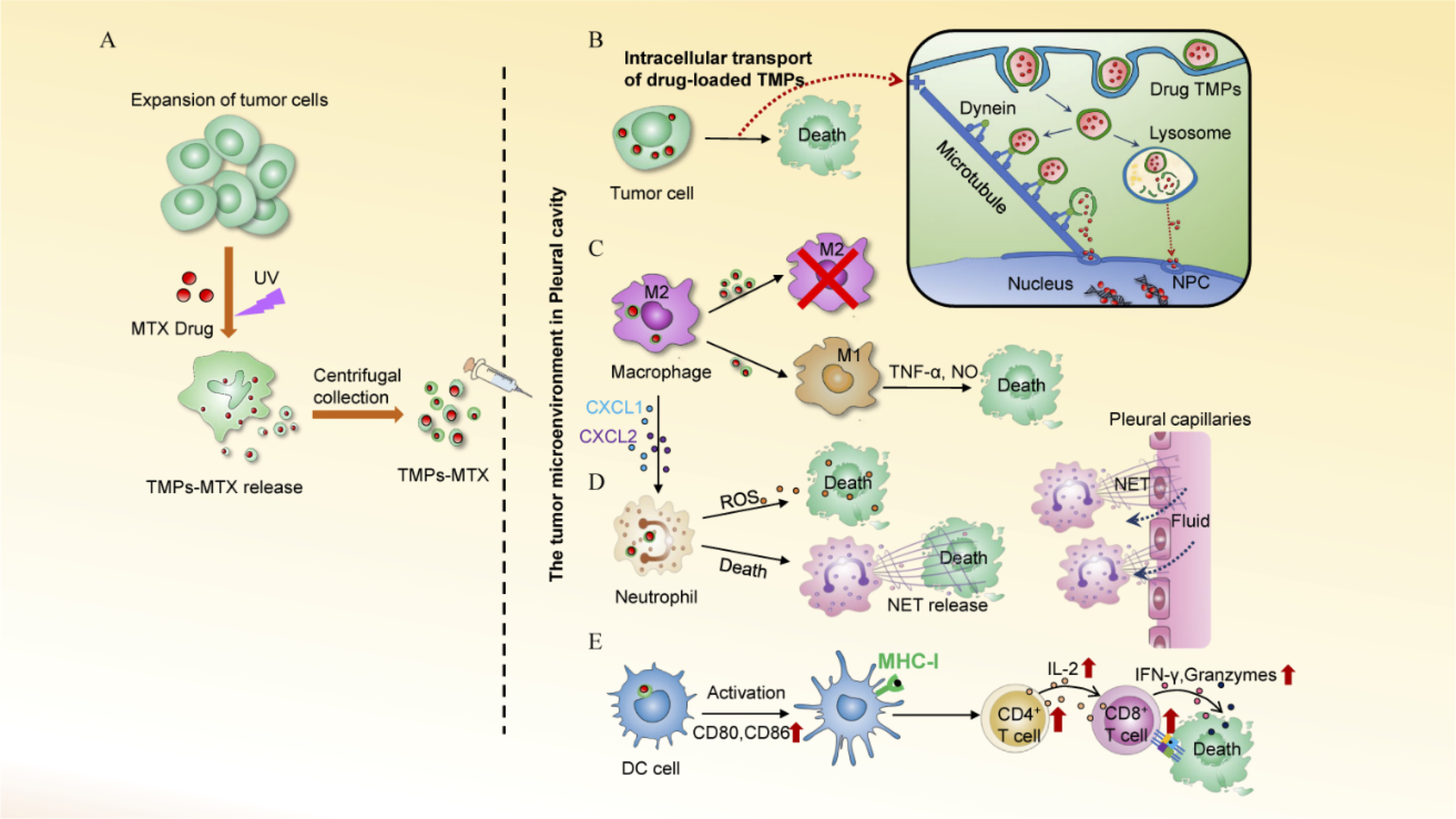

Supplement: Supplementary file 1 [file Image_1.jpeg]
