# Supplementary material for: Intrapleural infusion of tumor cell-derived microparticles packaging methotrexate or saline combined with pemetrexed-cisplatin chemotherapy for the treatment of malignant pleural effusion in advanced non-squamous non-small cell lung cancer: A double-blind, randomized, placebo-controlled study
Source: Front Immunol. 2022 Oct 5;13:1002938. doi: 10.3389/fimmu.2022.1002938 (PMC9580337; doi:10.3389/fimmu.2022.1002938)
Supplement: Supplementary file 2 [file DataSheet_1.docx]

**Supplementary Table 1. Comparison of blood tumor markers at different follow-up times between the two groups**

| **Time** | **Blood tumor markers (median)** | **Microparticles group (n = 40)** | **Placebo group (n = 39)** | ***P*-value** |
| --- | --- | --- | --- | --- |
| Before treatment | CEA (μg/L) | 22.63 | 19.50 | 0.5543 |
|  | CYFRA21-1(ng/mL) | 4.59 | 4.99 | 0.3837 |
|  | CA125 (U/mL) | 118.60 | 97.50 | 0.2831 |
|  | CA19-9 (U/mL) | 7.63 | 8.72 | 0.2871 |
| After treatment (16 ± 1 days after chemotherapy) | CEA (μg/L) | 18.05 | 11.89 | 0.2171 |
|  | CYFRA21-1(ng/mL) | 2.64 | 3.03 | 0.2376 |
|  | CA125 (U/mL) | 69.80 | 85.20 | 0.5824 |
|  | CA19-9 (U/mL) | 8.80 | 8.60 | 0.3188 |
| Follow-up period  (28 ± 2 days after the end of treatment) | CEA (μg/L) | 11.58 | 9.38 | 0.3233 |
|  | CYFRA21-1(ng/mL) | 2.48 | 2.28 | 0.4468 |
|  | CA125 (U/mL) | 36.10 | 56.90 | 0.6566 |
|  | CA19-9 (U/mL) | 14.87 | 9.80 | 0.6702 |

**Supplementary Table 2. Rivalta test of pleural effusion at different follow-up times**

| **Time** | **Rivalta test** | **Microparticles group n (%)** | **Placebo group n (%)** | ***P*** |
| --- | --- | --- | --- | --- |
| Before treatment | Normal | 4 (10) | 4 (10) | 0.4609 |
|  | Abnormal | 30 75) | 31 (79) |  |
| After treatment (10±1 days after chemotherapy) | Normal | 5 (27.78) | 10 (31.25) | 0.7970 |
|  | Abnormal | 13 (72.22) | 22 (68.75) |  |
| After treatment (16±1 days after chemotherapy) | Normal | 2 (14.29) | 7 (31.82) | 0.4315 |
|  | Abnormal | 12 (85.71) | 15 (68.18) |  |
| Follow-up period  (28±2 days after the end of treatment.) | Normal | 0 (0.00) | 3 (60.00) | 0.0350 |
|  | Abnormal | 8 (100.00) | 2 (40.00) |  |

**Supplementary Table 3. Biomarkers of pleural effusion at different follow-up times**

| **Time** | **Biomarkers (median)** | **Microparticles group** | **Placebo group** | ***P*-value** |
| --- | --- | --- | --- | --- |
| Before treatment | Total protein (g/L) | 42.10 | 39.85 | 0.7588 |
|  | Glucose (mmol/L) | 5.98 | 5.57 | 0.7381 |
|  | LDH (U/L) | 342.50 | 413.20 | 0.6722 |
|  | CEA (μg/L) | 130.30 | 242.44 | 0.9197 |
| After treatment (10 ± 1 days after chemotherapy) | Total protein (g/L) | 37.00 | 32.80 | 0.1062 |
|  | Glucose (mmol/L) | 5.94 | 6.06 | 0.3353 |
|  | LDH (U/L) | 462.00 | 365.00 | 0.0771 |
|  | CEA (μg/L) | 109.11 | 97.05 | 0.5703 |
| After treatment (16 ± 1 days after chemotherapy) | Total protein (g/L) | 37.90 | 30.70 | 0.1005 |
|  | Glucose (mmol/L) | 5.54 | 5.67 | 0.2101 |
|  | LDH (U/L) | 691.00 | 293.00 | 0.1235 |
|  | CEA (μg/L) | 57.75 | 86.28 | 0.6124 |
| Follow-up period  (28 ± 2 days after the end of treatment) | Total protein (g/L) | 43.40 | 44.10 | 0.7882 |
|  | Glucose (mmol/L) | 3.99 | 5.94 | 0.3008 |
|  | LDH (U/L) | 333.00 | 344.80 | 0.5751 |
|  | CEA (μg/L) | 399.40 | 114.78 | 0.2819 |

LDH, lactate dehydrogenase.
